# Intake of dietary flavonoids and incidence of ischemic heart disease in the Danish Diet, Cancer, and Health cohort

**DOI:** 10.1038/s41430-022-01226-y

**Published:** 2022-10-25

**Authors:** Benjamin H. Parmenter, Frederik Dalgaard, Kevin Murray, Guillaume Marquis-Gravel, Aedín Cassidy, Catherine P. Bondonno, Joshua R. Lewis, Kevin D. Croft, Cecilie Kyrø, Gunnar Gislason, Augustin Scalbert, Anne Tjønneland, Kim Overvad, Jonathan M. Hodgson, Nicola P. Bondonno

**Affiliations:** 1 School of Biomedical Sciences, University of Western Australia, Royal Perth Hospital, Perth, WA Australia; 2 Nutrition & Health Innovation Research Institute, School of Medical and Health Sciences, Edith Cowan University, Perth, WA Australia; 3 Department of Cardiology, Herlev & Gentofte University Hospital, Copenhagen, Denmark; 4 School of Population and Global Health, University of Western Australia, Perth, WA Australia; 5 Montreal Heart Institute, Université de Montréal, Montréal, QC Canada; 6 Institute for Global Food Security, Queen’s University, Belfast, Northern Ireland; 7 Medical School, University of Western Australia, Perth, WA Australia; 8 Centre for Kidney Research, School of Public Health, The University of Sydney, Sydney, NSW Australia; 9 Danish Cancer Society Research Center, Copenhagen, Denmark; 10 The National Institute of Public Health, University of Southern Denmark, Odense, Denmark; 11 The Danish Heart Foundation, Copenhagen, Denmark; 12 International Agency for Research on Cancer, Lyon, France; 13 Department of Public Health, Faculty of Health and Medical Sciences, University of Copenhagen, Copenhagen, Denmark; 14 Department of Public Health, Aarhus University, Aarhus, Denmark

**Keywords:** Epidemiology, Risk factors, Coronary artery disease and stable angina

## Abstract

**Background/Objectives:**

Few studies have investigated the association between dietary flavonoid intake, including all major subclasses, and the long-term risk of ischemic heart disease (IHD). We examined whether dietary flavonoid intake associated with IHD incidence, assessing the possible modifying role of sex and smoking, in participants from the Danish Diet, Cancer, and Health study.

**Subjects/Methods:**

In a cohort study design, 54,496 adults (46.8% male), aged 50–64 years, without a history of IHD, were followed for up to 23 years. Habitual dietary flavonoid intake was estimated from food frequency questionnaires using Phenol-Explorer. Incident cases of IHD were identified within Danish nationwide health registries. Restricted cubic splines in Cox proportional hazards models were used to examine associations between flavonoid intake and IHD risk.

**Results:**

During follow-up, 5560 IHD events were recorded. No overall association was seen between total flavonoid intake, nor any subclass, and IHD, following adjustment for demographics, lifestyle, and dietary confounders. Stratified by sex and smoking status, higher intakes of specific subclasses associated with lower IHD risk among ever-smokers [Q5 vs. Q1 flavonols HR (95% CI): 0.90 (0.82, 0.99); flavanol oligo+polymers: 0.88 (0.80, 0.97)], but not among never-smokers, nor either sex specifically.

**Conclusions:**

While we did not find clear evidence that higher habitual dietary flavonoid intake was associated with lower IHD risk, these results do not exclude the possibility that certain subclasses may have a protective role in prevention of IHD among population sub-groups; this was evident among smokers, who are at a higher risk of atherosclerosis.

## Introduction

Ischemic heart disease (IHD) is the leading cause of death globally, accounting for over 9 million fatalities per annum (~16% of total mortality) [1, 2]. Characterized by stenosed coronary arteries, IHD is mostly a consequence of atherosclerosis, which develops and progresses often over decades, prior to an acute coronary event of angina, myocardial infarction or its other pathological sequelae [3, 4]. Owing to their demonstrated beneficial effects on risk factors for atherosclerosis, flavonoids—bioactive compounds found in foods such as tea, cocoa, nuts, wine, fruits, and vegetables—may play a role in the prevention of IHD [5–7]. Evidence from RCTs suggests that flavonoid-rich food can reduce blood pressure, improve endothelial function, mitigate dyslipidaemia, lower arterial stiffness, and lessen inflammation [8–10]. Previous cohort studies have examined the association of flavonoid intake with IHD, and recent meta-analyses of these cohorts indicate that higher intakes of the flavonol, anthocyanin and flavanol flavonoid subclasses are associated with a significantly lower risk of IHD [11–13]. However, more than two-thirds of prior investigations only examined one or two flavonoid subclasses, as many were conducted prior to the development of comprehensive flavonoid-food composition databases [7]. Therefore, investigation of IHD outcomes associated with intake of the other flavonoid subclasses, such as flavones, flavanones and flavanol oligo+polymers as well as total flavonoid intake is needed. In our prior research, we found higher intakes of flavonoids were associated with a lower risk of atherosclerotic cardiovascular diseases including ischemic stroke [14], peripheral artery disease [15], and total atherosclerotic cardiovascular disease (CVD) [16]. Thus, we hypothesize, that higher habitual dietary flavonoid consumption will also be associated with a lower risk of IHD, the primary contributor to CVD events. We have also previously observed effect modification by smoking status, such that smokers appear to benefit more from higher flavonoid intakes [14–16]. Consequently, we further hypothesise, that the associations between flavonoid intake and IHD will be present in smokers, who are at a higher risk of atherosclerosis. As such, in the present study, we examined the relationship between total flavonoid and flavonoid subclass intake with IHD risk in the large Danish Diet, Cancer, and Health cohort.

## Methods

### Study design and population

The details of the Danish Diet, Cancer, and Health study, its procedures and population characteristics, have been reported elsewhere [17]. Briefly, between 1993 and 1997, 57,053 male and female adults, aged 50–64 years old, who lived in the Copenhagen and Aarhus areas of Denmark were enrolled into this cohort [17]. Blood samples, anthropometric measures and medical history were obtained at study entry as were data on diet and lifestyle. Participants who reported a history of IHD (*n* = 1557), percutaneous coronary intervention (*n* = 6), coronary artery bypass graft (*n* = 8), or cancer diagnosis before baseline (*n* = 574) were excluded, as were participants with missing or extreme covariates (*n* = 211), incomplete diet data (*n* = 51), or implausible energy intakes (<500 or >5000 kcal/day; *n* = 150), leaving 54,496 participants for analysis (Supplementary Fig. 1). The study was approved by the Danish Data Protection Agency (Ref no 2012-58-0004 I-Suite nr: 6357, VD-2018-117) and all participants provided informed consent. Requests to access the dataset may be sent to the Diet, Cancer and Health Steering Committee at the Danish Cancer Society.

### Assessment of diet and flavonoid intake

Habitual dietary intake was estimated using a 192-item food frequency questionnaire (FFQ) which was specifically designed and validated to assess food consumption in Denmark [18, 19]. Daily average intakes of nutrients and flavonoids were estimated from the FFQ using the software programme FoodCalc and the Phenol-Explorer food composition database, as described previously [16, 17, 20, 21]. Briefly, intakes of individual nutrients and flavonoid compounds were calculated by multiplying the frequency of consumption of relevant foods by their nutrient and flavonoid contents for pre-specified portion sizes. Individual flavonoid compounds, without hydrolysis of glycosides or esters, were summed into the following subclasses: flavonols, flavanol monomers, flavanol oligo + polymers (including theaflavins), flavanones, flavones, anthocyanins, isoflavones, dihydrochalcones, dihydroflavonols and chalcones. Total flavonoid intake was estimated by the summation of all subclasses. Exposures in this analysis included total flavonoids and all subclasses except isoflavones, dihydrochalcones, dihydroflavonols and chalcones as their average daily intake was very low in this cohort (<5 mg/day).

### Outcomes ascertainment

Danish nationwide registries were used to identity and record cases of IHD, classified until 1993 according to the International Classification of Diseases (ICD) 8^th^ revision (ICD-8) and thereafter, according to the 10th revision (ICD-10) [22]. The primary outcome was defined as all first-time IHD events (IHD-coded hospitalizations and deaths) using ICD-10 codes I20–I25 and ICD-8 codes 41009-41499. Unknown deaths (which may or may not be IHD-related-related) were not considered in the primary outcome. Hospitalizations for IHD were extracted from The Danish National Patient Register, a national collection of hospital discharge information, and death data was obtained from The Danish Register of Causes of Death. The ICD codes for myocardial infarction hospitalizations have a positive predictive value of >90% [23].

### Validated case analysis

To verify the registry-based outcomes, we re-examined associations only using cases that have been previously medically reviewed and validated for first-time acute myocardial infarction (ICD-10: I21), with a follow-up time frame of 19 years between February 1994 and April 2013 [24].

### Covariates

Information on lifestyle factors and medical history including sex, age, anthropometry, physical activity, education, smoking habits, alcohol consumption, medication use and diet were obtained from the baseline assessment. To assess socioeconomic status, each participant’s average annual income over 5-years (defined as household income after taxation and interest, for the value of the Danish currency in 2015) was used. For hypertension and diabetes mellitus, self-reported data were used due to the underreporting of these diagnosis in the Danish National Patient Register (DNPR) [23]. Comorbidities of peripheral artery disease, ischemic stroke, chronic kidney disease, chronic obstructive pulmonary disease, and atrial fibrillation were identified by ICD codes dated prior to enrollment (Supplementary Table 1).

### Statistical analysis

The association between flavonoid intake (exposures) and IHD risk was modeled using restricted cubic splines within Cox proportional hazards models. In all analyses, exposures were treated as continuous variables with hazard ratios (HRs) and confidence intervals (95% CI) extracted and reported at the median intake in each quintile, with the first quintile median as the reference point. Time-to-event was calculated for each participant from enrollment until the end of follow-up (August, 2017), an IHD event, death, or emigration (loss to follow-up [0.49% of cohort]), whichever occurred first. Given the etiological focus, all deaths were censored rather than treated as a competing risk [25]. Proportional hazards assumptions were tested by visually inspecting log-log plots of the survival function versus time, with no violation found. For all analyses, three models of adjustment were used: Model 1) minimally adjusted: age (years) and sex (male/female); Model 2) multivariable-adjusted: age, sex, BMI (kg/m^2^), smoking status (current/former/never), physical activity (total daily metabolic equivalent), alcohol intake (g/d), education (≤7/8–10/≥11 years), socioeconomic status (income), aspirin use, antihypertensive medication use and statin use; Model 3) multivariable-adjusted including potential dietary confounders; covariates in Model 2 plus intakes (g/d) of fish, red meat, processed meat, whole-grain products, processed grains, polyunsaturated fatty acids, monounsaturated fatty acids, saturated fatty acids and energy (kJ/d). In secondary analyses, we stratified the cohort by sex (male/female) and smoking status (ever-smoker/never-smoker) to explore associations in distinct subgroups with differing IHD risk factors. Analyses stratified by smoking status were additionally adjusted for pack-years of smoking duration in the relevant sub-group. All analyses were undertaken using STATA/IC 14.2 (StataCorp LLC) and R statistics (R Core Team, 2021) [26]. Statistical significance was set at *P* ≤ 0.05 (two-tailed).

## Results

During a median [IQR] of 20.8 [18.5–21.7] years (maximum 23 years) follow-up, 5560 cases of IHD death or first-time hospitalization occurred. A total of 11,195 participants died from causes other than IHD and without a prior diagnosis of IHD. The median age at baseline was 56 years, females constituted 53.2% of the sample and 35.9% were current smokers (Table 1). Participants had a median [IQR] total flavonoid intake of 497 [288–807] mg/day which was driven mostly by intakes of flavanol oligo+polymers and flavanol monomers. Participants with higher flavonoid intakes tended to smoke less, exercise more, maintain a lower BMI, and have a higher education and income. Those consuming more flavonoids also tended to eat more fish, fiber, whole-grain products, fruits, and vegetables, and eat less red and processed meat (Table 1).

**Table 1:** Baseline characteristics of study population^a^.

	Total population *n* = 54,496	Total flavonoid intake quintiles				
		Q1 *n* = 10,900	Q2 *n* = 10,899	Q3 *n* = 10,899	Q4 *n* = 10,899	Q5 *n* = 10,899
Demographic characteristics						
Total flavonoid intake (mg/d)^c^	496.53 (288–807)	174 (6–252)	322 (252–396)	497 (396–603)	728 (603–912)	1205 (912–3552)
Sex (male)	25,477 (46.8)	6202 (56.9)	5498 (50.4)	5100 (46.8)	4755 (43.6)	3922 (36.0)
Age (years)	56 [52–60]	56 [52–60]	56.00 [52–60]	56.00 [52–60]	56.00 [52–60]	55.00 [52–60]
BMI (kg/m^2^)	25.5 [23.2–28.2]	26.1 [23.7–28.8]	25.8 [23.6–28.4]	25.5 [23.3–28.2]	25.3 [23.1–27.8]	24.8 [22.7–27.3]
MET score	56.50 [37.0–84.5]	51.0 [32.5–78.0]	55.5 [36.3–84.0]	57.3 [38.4–84.9]	58.3 [38.5–87.0]	60.0 [40.0–88.5]
Smoking status						
Never	19,423 (35.6)	2723 (25.0)	3705 (34.0)	3955 (36.3)	4375 (40.1)	4665 (42.8)
Former	15,515 (28.5)	2590 (23.8)	2912 (26.7)	3117 (28.6)	3470 (31.8)	3426 (31.4)
Current	19,558 (35.9)	5587 (51.3)	4282 (39.3)	3827 (35.1)	3054 (28.0)	2808 (25.8)
Education						
≤7 years	17,707 (32.5)	4941 (45.3)	4099 (37.6)	3442 (31.6)	2902 (26.6)	2323 (21.3)
8–10 years	25,228 (46.3)	4780 (43.9)	5155 (47.3)	5237 (48.1)	5177 (47.5)	4879 (44.8)
≥11 years	11,536 (21.2)	1174 (10.8)	1642 (15.1)	2216 (20.3)	2814 (25.8)	3690 (33.9)
Mean household income^b^						
≤394,700 DKK/year	13,408 (24.6)	3216 (29.5)	2655 (24.4)	2611 (24.0)	2494 (22.9)	2432 (22.3)
394,701–570,930 DKK/year	13,539 (24.8)	3158 (29.0)	2905 (26.7)	2624 (24.1)	2509 (23.0)	2343 (21.5)
570,931–758,297 DKK/year	13,709 (25.2)	2851 (26.2)	2964 (27.2)	2821 (25.9)	2557 (23.5)	2516 (23.1)
>758,297 DKK/year	13,840 (25.4)	1675 (15.4)	2375 (21.8)	2843 (26.1)	3339 (30.6)	3608 (33.1)
Comorbidities						
Hypertensive	8580 (15.7)	1685 (15.5)	1765 (16.2)	1736 (15.9)	1709 (15.7)	1685 (15.5)
Hypercholesterolemic	3585 (6.6)	744 (6.8)	720 (6.6)	742 (6.8)	734 (6.7)	645 (5.9)
Diabetes	1068 (2.0)	258 (2.4)	200 (1.8)	225 (2.1)	186 (1.7)	199 (1.8)
Peripheral artery disease	404 (0.7)	139 (1.3)	91 (0.8)	66 (0.6)	48 (0.4)	60 (0.6)
Ischemic stroke	673 (1.2)	178 (1.6)	137 (1.3)	128 (1.2)	112 (1.0)	118 (1.1)
Atrial fibrillation	226 (0.4)	43 (0.4)	44 (0.4)	50 (0.5)	39 (0.4)	50 (0.5)
COPD	778 (1.4)	199 (1.8)	168 (1.5)	142 (1.3)	144 (1.3)	125 (1.1)
CKD	188 (0.3)	35 (0.3)	31 (0.3)	42 (0.4)	40 (0.4)	40 (0.4)
Medication use						
Insulin treated	347 (0.6)	73 (0.7)	61 (0.6)	78 (0.7)	69 (0.6)	66 (0.6)
Antihypertensive medication use	6427 (11.8)	1247 (11.4)	1344 (12.3)	1300 (11.9)	1266 (11.6)	1270 (11.7)
Statin use	702 (1.3)	153 (1.4)	152 (1.4)	145 (1.3)	136 (1.2)	116 (1.1)
HRT						
Never	15,785 (29.0)	2592 (23.8)	3009 (27.6)	3228 (29.6)	3241 (29.7)	3715 (34.1)
Current	8714 (16.0)	1288 (11.8)	1551 (14.2)	1676 (15.4)	1973 (18.1)	2226 (20.4)
Former	4489 (8.2)	809 (7.4)	837 (7.7)	888 (8.1)	924 (8.5)	1031 (9.5)
NSAID	17,484 (32.3)	3386 (31.3)	3397 (31.4)	3502 (32.3)	3509 (32.3)	3690 (34.1)
Aspirin use	6704 (12.3)	1292 (11.9)	1297 (11.9)	1352 (12.4)	1324 (12.1)	1439 (13.2)
Dietary characteristics						
Energy (kj)	9493 [7853–11,363]	8603 [7030–10,396]	9245 [7702–10,995]	9740 [8127–11,583]	9927 [8319 –11,815]	9919 [8255–11,875]
Saturated FA (g/d)	31 [24–39]	29 [23–37]	31 [24–39]	32 [25–40]	32 [25–41]	32 [24–41]
Polyunsaturated FA (g/d)	13 [10–17]	12 [9–16]	13 [10–17]	14 [10–18]	14 [11–18]	14 [10–18]
Monounsaturated FA (g/d)	27 [21–35]	26 [20–34]	27 [21–35]	28 [22–35]	28 [22–35]	27 [21–34]
Total fish intake (g/d)	38 [25–55]	33 [22–48]	38 [25–54]	40 [27–57]	41 [28–59]	40 [27–56]
Red meat intake (g/d)	78 [56–107]	80 [57–108]	81 [59–109]	80 [58–110]	78 [56–107]	72 [52–99]
Processed meat intake (g/d)	24 [14–40]	28.3 [17–45]	26 [15–42]	25 [14–40]	23 [14–38]	20 [11–34]
Whole-grain product intake (g/d)	128 [87–175]	116 [72–165]	123 [84–171]	126 [86–173]	135 [97–181]	144 [103–192]
Processed grain intake (g/d)	46 [29–72]	45 [27–80]	46 [29–73]	47 [30–72]	46 [30–70]	45 [29–68]
Fruit intake (g/d)	172 [95–281]	88 [44–141]	162 [98–238]	193 [114–301]	224 [140–360]	240 [141–389]
Apples and pears (g/d)	54 [10–98]	10 [4–54]	54 [10–98]	54 [18–125]	54 [18–125]	54 [18–125]
Orange (g/d)	14 [3–43]	8 [2–14]	8 [3–43]	14 [8–43]	14 [8–43]	14 [8–43]
Vegetable intake (g/d)	162 [105–231]	114 [72–170]	150 [100–212]	168 [114–235]	185 [127–254]	196 [135–272]
Chocolate (g/d)	4 [2–7]	2 [1–4]	4 [2–7]	4 [2–7]	4 [2–7]	4 [2–21]
Alcohol intake (g/d)	13 [6–31]	11 [3–23]	13 [6–25]	15 [6–33]	14 [7–32]	13 [6–32]
Wine (ml/d)	54 [10–98]	18 [10–54]	54 [10–98]	54 [10–125]	54 [18–125]	54 [18–125]
Black tea (ml/d)	86 [3–500]	3 [0–16]	16 [3–86]	86 [7–200]	500 [157–500]	900 [500–1300]

*CKD* chronic kidney disease, *COPD* chronic obstructive pulmonary disease, *BMI* body mass index, *DKK* Danish Krone, *FA* fatty acids, *HRT* hormone replacement therapy, *MET* metabolic equivalent, *NSAID* non-steroidal anti-inflammatory.

^a^Data expressed as median [IQR] or n (%), unless otherwise stated.

^b^The categories of household income in United States Dollar (USD) equivalents is approximately ≤61,985/year; 61,986–89,661/year; 89,662–119,086/year; >119,087/year.

^c^Median; range in parentheses (all such values). Intake quintiles are mutually exclusive.

### Associations between habitual flavonoid intake and ischemic heart disease incidence

**Fig. 1:**
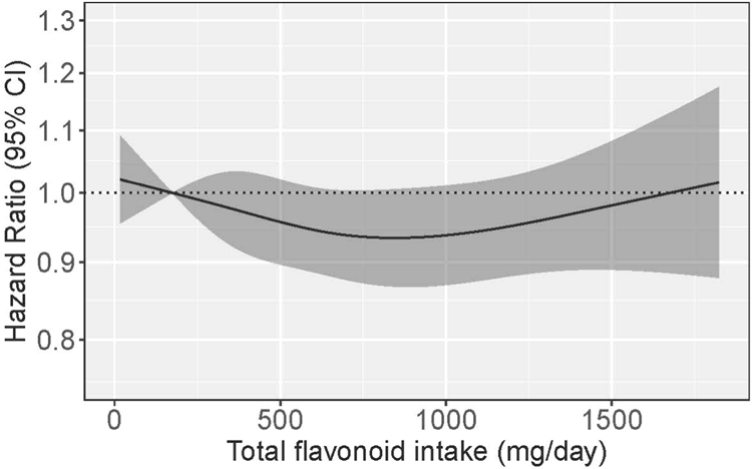
Cubic spline curves describing the association between total flavonoid intake and ischemic heart disease incidence (*n* = 5560) among participants of the Danish Diet, Cancer and Health cohort. Hazard ratios are based on Cox proportional hazards models adjusted for age, sex, BMI, smoking status, physical activity, alcohol intake, education, socioeconomic status (income), aspirin use, antihypertensive medication use and statin use and are comparing the specific level of flavonoid intake (horizontal axis) to the median intake for participants in the lowest intake quintile.

**Fig. 2:**
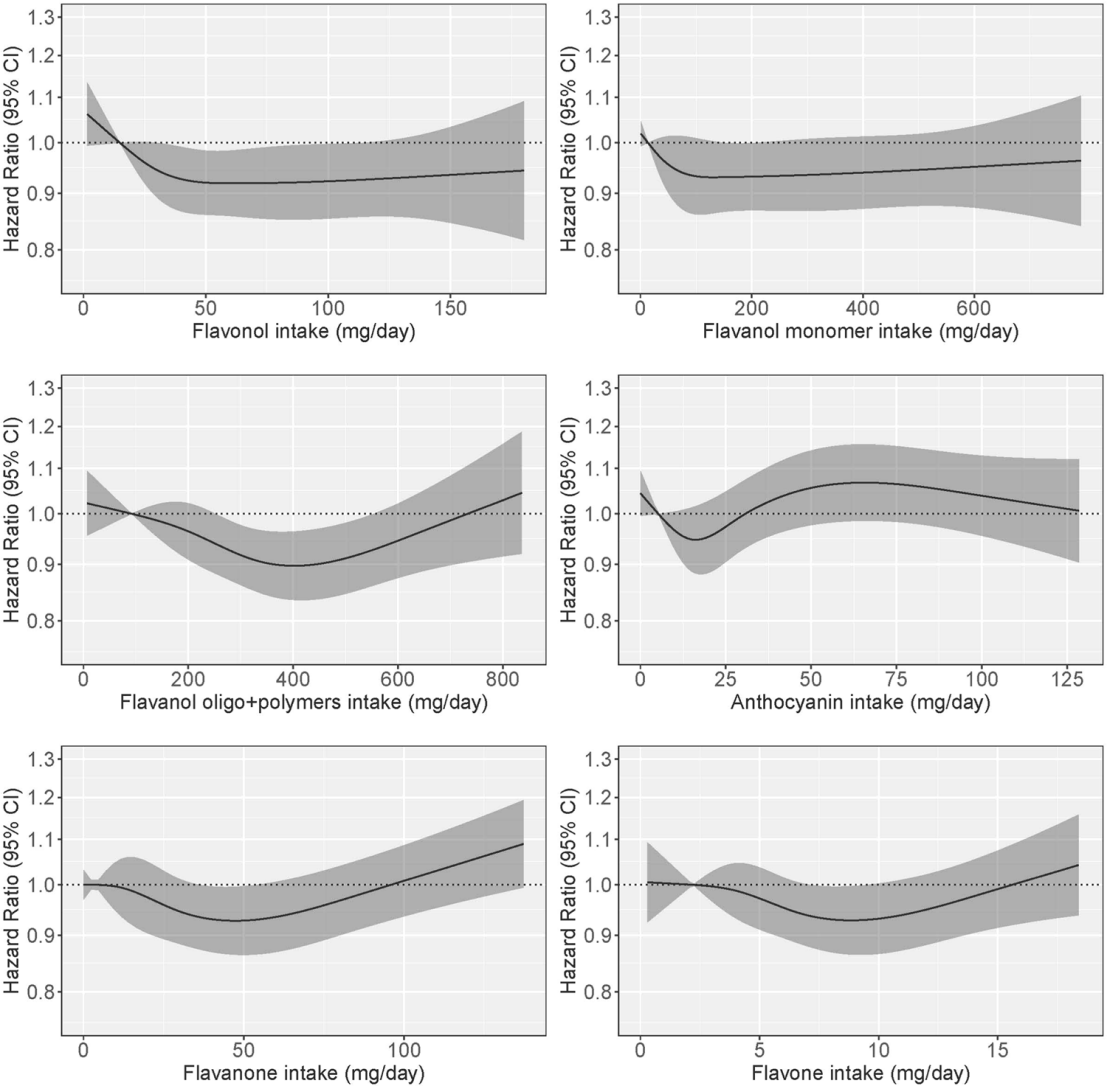
Cubic spline curve describing the associations between flavonoid subclass intakes (mg/day) and ischemic heart disease incidence (*n* = 5560) among participants of the Danish Diet, Cancer and Health cohort. Hazard ratios are based on Cox proportional hazards models adjusted for age, sex, BMI, smoking status, physical activity, alcohol intake, education, socioeconomic status (income), aspirin use, antihypertensive medication use and statin use and are comparing the specific level of flavonoid intake (horizontal axis) to the median intake for participants in the lowest intake quintile.

For intakes of total flavonoids beyond quintile 1, we observed a trend towards a lower risk of IHD, however this was not significant in models adjusted for demographics and lifestyle characteristics (Model 2; Table 2; Fig. 1) or dietary confounders (Model 3; Table 2). Of the individual flavonoid subclasses assessed (including flavonols, flavanol monomers, flavanol oligo+polymers, anthocyanins, flavanones and flavones), although some subclasses (flavanol oligo+polymers and flavonols) were associated with a lower IHD risk in models adjusted for demographics and lifestyle factors, no clear or significant associations were present for any subclass, when models were further adjusted for dietary confounders (Model 2; Model 3; Table 2; Fig. 2).

**Table 2:** Hazard ratios of first-time ischemic heart disease by quintiles of flavonoid intake^a^.

	Flavonoid intake quintiles				
	Q1 (*n* = 11,034)	Q2 (*n* = 11,034)	Q3 (*n* = 11,033)	Q4 (*n* = 11,034)	Q5 (*n* = 11,034)
Total flavonoids					
No. events	1392	1172	1100	1008	888
Intake (mg/d)^b^	174 (6–252)	322 (252–396)	497 (396–603)	728 (603–912)	1205 (912–3552)
HR (95% CI)					
Model 1	Ref.	0.87 (0.83, 0.91)	0.77 (0.73, 0.83)	0.72 (0.67, 0.77)	0.70 (0.65, 0.76)
Model 2	Ref.	0.98 (0.93, 1.03)	0.96 (0.90, 1.02)	0.94 (0.87, 1.00)	0.95 (0.88, 1.03)
Model 3	Ref.	0.99 (0.94, 1.04)	0.97 (0.91, 1.04)	0.96 (0.89, 1.03)	0.98 (0.90, 1.06)
Flavonols					
No. events	1409	1198	1082	1008	863
Intake (mg/d)^b^	15 (0–21)	26 (21–32)	39 (32–50)	66 (50–83)	116 (83–251)
HR (95% CI)					
Model 1	Ref.	0.85 (0.81, 0.89)	0.76 (0.71, 0.81)	0.69 (0.65, 0.74)	0.68 (0.63, 0.73)
Model 2	Ref.	0.96 (0.91, 1.00)	0.93 (0.87, 0.99)	0.92 (0.86, 0.99)	0.93 (0.86, 1.00)
Model 3	Ref.	0.96 (0.92, 1.01)	0.94 (0.88, 1.01)	0.94 (0.87, 1.02)	0.96 (0.88, 1.04)
Flavanol monomers					
No. events	1433	1159	1104	994	870
Intake (mg/d)^b^	14 (0 –21)	30 (21–46)	67 (46–116)	261 (116–282)	474 (282–916)
HR (95% CI)					
Model 1	Ref.	0.91 (0.88, 0.94)	0.78 (0.73, 0.84)	0.71 (0.66, 0.76)	0.71 (0.66, 0.76)
Model 2	Ref.	0.98 (0.95, 1.01)	0.94 (0.88, 1.02)	0.93 (0.87, 1.01)	0.94 (0.88, 1.02)
Model 3	Ref.	0.99 (0.95, 1.02)	0.96 (0.90, 1.04)	0.96 (0.89, 1.04)	0.98 (0.91, 1.05)
Flavanol oligo + polymers					
No. events	1378	1239	1026	985	932
Intake (mg/d)^b^	92 (0–137)	180 (137–218)	256 (218–303)	360 (303–434)	538 (435–2254)
HR (95% CI)					
Model 1	Ref.	0.85 (0.81, 0.90)	0.76 (0.72, 0.81)	0.70 (0.65, 0.75)	0.69 (0.64, 0.74)
Model 2	Ref.	0.97 (0.92, 1.03)	0.94 (0.88, 1.00)	0.90 (0.84, 0.96)	0.92 (0.86, 0.99)
Model 3	Ref.	0.98 (0.93, 1.04)	0.95 (0.89, 1.02)	0.92 (0.85, 0.99)	0.94 (0.87, 1.02)
Anthocyanins					
No. events	1409	1198	1082	1008	863
Intake (mg/d)^b^	5 (0–10)	13 (10–17)	20 (17–24)	36 (24–53)	70 (53–397)
HR (95% CI)					
Model 1	Ref.	0.78 (0.74, 0.83)	0.71 (0.67, 0.76)	0.76 (0.71, 0.81)	0.83 (0.78, 0.90)
Model 2	Ref.	0.95 (0.90, 1.01)	0.95 (0.88, 1.03)	1.02 (0.95, 1.10)	1.07 (0.99, 1.16)
Model 3	Ref.	0.95 (0.90, 1.01)	0.96 (0.89, 1.03)	1.03 (0.95, 1.11)	1.08 (0.99, 1.17)
Flavanones					
No. events	1238	1127	1098	994	1103
Intake (mg/d)^b^	3 (0–6)	9 (6–13)	18 (13–26)	32 (26–49)	70 (49–564)
HR (95% CI)					
Model 1	Ref.	0.93 (0.89, 0.98)	0.87 (0.80, 0.93)	0.82 (0.77, 0.88)	0.84 (0.79, 0.91)
Model 2	Ref.	1.00 (0.95, 1.05)	0.98 (0.91, 1.06)	0.94 (0.88, 1.01)	0.95 (0.88, 1.02)
Model 3	Ref.	1.00 (0.96, 1.05)	0.99 (0.92, 1.07)	0.95 (0.88, 1.01)	0.95 (0.88, 1.02)
Flavones					
No. events	1201	1171	1085	1016	1087
Intake (mg/d)^b^	2 (0–3)	4 (3–4)	5 (4–6)	7 (6–9)	11 (9–51)
HR (95% CI)					
Model 1	Ref.	0.90 (0.86, 0.95)	0.84 (0.79, 0.90)	0.81 (0.76, 0.86)	0.84 (0.78, 0.90)
Model 2	Ref.	0.99 (0.94, 1.04)	0.97 (0.91, 1.03)	0.94 (0.88, 1.00)	0.94 (0.88, 1.01)
Model 3	Ref.	1.00 (0.94, 1.05)	0.98 (0.91, 1.05)	0.95 (0.88, 1.02)	0.96 (0.88, 1.04)

^a^Hazard ratios (95% CI) for first-time ischemic heart disease during 20.8 years of follow-up, obtained from restricted cubic splines in Cox proportional hazards models. Model 1 adjusted for age and sex; Model 2 adjusted for age, sex, BMI, smoking status, physical activity, pure alcohol intake, education, social economic status (income), aspirin use, antihypertensive medication use and statin use; Model 3 adjusted for all covariates in Model 2 plus intakes of fish, red meat, processed meat, whole-grain products, processed grains, polyunsaturated fatty acids, monounsaturated fatty acids, saturated fatty acids and energy.

^b^Median; range in parentheses (all such values).

### Validated case analysis

Using only validated first-time cases, 2613 participants were hospitalized or died from acute myocardial infarction. Although a lower risk of IHD was seen for higher intakes of total flavonoids, the association was not significant (Model 2; Supplementary Fig. 2).

### Associations between habitual flavonoid intake and ischemic heart disease incidence stratified by sex and smoking status

Stratified by sex, and after adjustment for demographic and lifestyle factors, higher total flavonoid intake was significantly associated with lower IHD risk in males but not females (Model 2; Supplementary Table 2). However, with additional adjustment for dietary confounders, no significant association was present in either sex (Model 3; Supplementary Table 2). Of the individual flavonoid subclasses assessed, following adjustment for demographic and lifestyle factors, higher intakes of flavonols, flavanol monomers and flavanol oligo+polymers were significantly associated with lower IHD risk in males but not females (Model 2; Supplementary Table 2). When models were further adjusted for dietary covariates there was no compelling evidence of a lower IHD risk in either sex, with higher intakes, of any subclass (Model 3; Supplementary Table 2).

Stratified by smoking status, and after adjustment for demographic and lifestyle factors, higher total flavonoid intakes were significantly associated with lower IHD risk in ever-smokers but not never-smokers (Model 2; Supplementary Table 3). However, following additional adjustment for dietary confounders, a significant lower risk of IHD was not seen in ever-smokers nor never-smokers (Model 3; Supplementary Table 3). Of the individual flavonoid subclasses, among those who had never smoked, no significant inverse associations were seen (Model 2; Model 3; Supplementary Table 3). Among smokers, higher intakes (Q5 vs Q1) of flavonols, flavanol monomers and flavanol oligo+polymers were significantly associated with a 13% [HR (95% CI): 0.87 (0.79, 0.95)], 11% [0.89 (0.82, 0.97)], and 13% [0.87 (0.80, 0.94)] lower risk of IHD, respectively, following multivariable adjustment for demographics and lifestyle (Model 2; Supplementary Table 3). With additional adjustment for dietary covariates, significant beneficial associations (Q5 vs Q1) remained for flavonols [HR (95% CI): 0.90 (0.82, 0.99)] and flavanol oligo+polymers [HR (95% CI): 0.88 (0.8, 0.97)].

## Discussion

In this prospective cohort study, of 54,496 Danish adults, aged 50–64 years, who were followed for up to 23 years, we did not observe clear associations between intakes of total flavonoids or flavonoid subclasses with IHD risk. Nor did we observe compelling evidence of effect modification by sex, such that higher flavonoid intakes associated with lower IHD risk in one sex, but not the other. On the other hand, when we stratified our sample by smoking status, we saw that higher intake of flavonols and flavanol oligo+polymers was associated with lower IHD risk among ever-smokers, but not never-smokers. Thus, while the findings do not support a strong benefit of flavonoid intake on IHD risk, they also do not exclude the possibility that flavonoids may have a modest protective role in IHD, particularly for current or former smokers.

It is of particular relevance that we have observed in our previous investigations of the relationship between flavonoid intake and CVD in the Danish, Diet, Cancer and Health Study, that higher intakes of flavonoids was clearly associated with a lower risk of total atherosclerotic CVD and, more specifically, peripheral artery disease and ischemic stroke in the total population [14–16]. In the present study, flavonol and flavanol oligo+polymer intake associated with lower IHD risk in models adjusted for demographic and lifestyle characteristics; however, no significant associations were present in models additionally adjusted for dietary confounders. This suggests that the observed associations between flavonoid intake and IHD risk may be a consequence of differing underlying dietary patterns. Given our promising prior results, and the common etiology of atherosclerosis in CVD, it is thus surprising that the present analysis did not find clear evidence of associations between flavonoids and IHD. Similar seemingly disparate findings have previously been observed. For example, in the Health Professionals Follow-Up study, higher flavanone consumption strongly associated with a lower risk of stroke but not myocardial infarction, and in the NutriNet-Santé Cohort, higher intakes of flavonols and catechins were associated with a lower risk of stroke but not coronary heart disease [27, 28]. The reasons for the differing results between CVD types are less certain, yet may allude to differences in the underlying pathology and the relative contributions of flavonoids on these pathologies.

Ischemic stroke, PAD and IHD are often discussed as diseases of the same entity: atherosclerotic arterial disease. The pathophysiology of these diseases involve many common risk pathways, but it is possible that inflammation, hyperlipidaemia and hypertension, along with other risk factors, contribute differentially to atherosclerosis of the cerebral, coronary and peripheral vascular beds. In fact, growing evidence does suggest a heterogeneous impact of atherosclerotic risk factors on different vascular regions. Several cohort studies have reported higher blood pressure is a stronger predictor of ischemic stroke than IHD and that elevated blood lipids appear to play a more important role in the development of IHD than ischemic stroke [29–31]. Consequently, the effect of reducing blood pressure or blood lipids may be more pronounced on the CVD type with which the risk factor is more strongly associated [32]. For flavonoids, the evidence to date appears to indicate a stronger anti-hypertensive action, than hypo-lipidemic action [8, 13]. As a result, the habitual intake of flavonoids may more strongly associate with ischemic stroke than IHD, which could explain our limited findings. A growing body of data also reports hypercoagulability is a stronger risk factor for ischemic stroke than for myocardial infarction [33]. Flavonoids appear to modulate key events in the pathogenesis of thrombosis via multiple mechanisms, such as reducing platelet activation, enhancing NO production and blocking TxA_2_ receptors [34]. As such, the anti-thrombotic activities of flavonoids may be further contributing to divergent associations among CVD types.

It is also known, that even though IHD, cerebrovascular disease, and PAD all share atherogenic pathophysiology, the mechanisms underlying the occurrence of acute events largely differ. Indeed, while progressive stenosis of carotid and of peripheral arteries primarily account for non-cardio-embolic ischemic strokes and for symptomatic PAD, most acute coronary syndromes are caused by atherosclerotic plaque ruptures at sites with only mild arterial narrowing [35]. It has been shown that coronary atherosclerotic plaque morphology is a more important prognostic factor for acute cardiac events than the degree of stenosis, with most events occurring in plaques with a phenotype characterized by a thin-cap fibroatheroma and a large plaque burden [36]. Therefore, the impact of flavonoids may address diseases in which the degree of arterial stenosis represents a stronger pathophysiological component.

In contrast to the results of our study, among previous cohort investigations of flavonoids, their subclasses and IHD, significant inverse associations have been observed [7]. Indeed, other cohort studies have observed evidence for a benefit of intake of certain subclasses on IHD risk, but not for cerebrovascular disease [37, 38]. These findings are difficult to reconcile, yet may be due to one or more underlying, unidentified, clinical (e.g., gut microbiome, genetic, or plaque-type differences) or methodological factors causing heterogeneity, among the various epidemiological studies. Indeed, we saw significant beneficial associations for all subclasses and total flavonoid intake in models adjusted for age and sex, but when socio-demographic data was added, nearly all these associations became statistically non-significant. It may therefore be, that the present study may not have failed to reproduce previously identified associations, instead the previous studies may have failed to identify all relevant confounders as well as the current investigation. Indeed, we found evidence of effect modification by smoking status, which is likely of consequence when examining results of different cohort studies.

In the present study, we observed effect modification by smoking status. The association between flavonoid intake and IHD was present in ever-smokers but not never-smokers. This is of note as in our previous studies of flavonoid intake and disease risk in the Danish Diet Cancer and Health cohort, those diseases for which smoking is a more influential risk factor, have all been more strongly associated with flavonoid intake. This extends to peripheral artery disease [15], abdominal aortic aneurysm [15], and chronic obstructive pulmonary disease [39]. There are several intriguing explanations for these observations. Smoking may modulate disease risk by amplifying pathogenic mechanisms common to smokers and non-smokers alike for which flavonoids show benefits, or smoking-related pathology may be initiated and/or mediated by differing/unique pathogenic mechanisms for which flavonoids show specific ameliorative effects. With regards to IHD specifically, cigarette smoking increases the risk at least partly through increasing systemic thrombotic propensity, oxidative stress and inflammation—mechanisms which appear attenuated via flavonoid intake [34, 40, 41]. Despite this, we have previously shown that smokers consuming high flavonoids still have a much higher risk of CVD than non-smokers consuming low flavonoids [14]. As such, public health campaigns for the primary prevention of IHD should prioritize smoking cessation.

This study has several limitations. While the cohort had a long follow up period, a high number of IHD cases, and complete data available on most participants, the assessment of demographics, lifestyle, and health habits at baseline alone means that we were unable to account for changes during follow-up. The methods of dietary assessment also lessen our certainty of the findings and further observational studies using biomarkers of flavonoid intake would be beneficial to determine if an association with IHD is seen using objective measures [42]. To this end, while Phenol Explorer represents the current state of the art for flavonoid-food composition databases, it is thought that this database will continue to be refined, and in years to come, more complete composition data, may become available; in this instance, updated analyses, testing our initial hypotheses, may be of value. It should be noted that several recent meta-analyses indicate higher intakes of flavonols, anthocyanins and flavanols are associated with lower IHD risk, and as such, we cannot rule out a potential beneficial relationship [11–13]. Indeed, in this population, tea, chocolate, wine, apples and pears appear to drive flavonoid intake, which is similar to other European and Western Countries, including the United States and Australia [6, 43, 44]. Even so, elucidating the impact of flavonoids on IHD via observational studies restricts our ability to infer causality or exclude the possibility of confounding. Moreover, the number of exposure-outcome combinations tested in our analysis increases the possibility of spurious findings. To this end, we did observe that females appeared to be at higher IHD risk with higher anthocyanin intakes, which we contend is unlikely. Although, this association may have arisen due to residual confounding from higher intakes of certain anthocyanin-bearing foods or beverages, which are not necessarily healthy when consumed in excess quantities, such as red wine or cordial-like fruit squash. Finally, considering the Danish population is more homogeneous than many other countries, the results of the present analysis may only be generalizable to other populations of similar age, race, health status and socioeconomic standing.

In conclusion, we did not observe clear associations between higher intakes of total flavonoids or flavonoid subclasses and lower IHD risk. However, we did observe a lower risk of IHD with higher intake of certain flavonoid subtypes among ever-smokers and thus we cannot rule out a benefit of flavonoid intake, especially among specific sub-groups at higher risk of atherosclerosis. These findings warrant additional research to further clarify the association between habitual dietary flavonoid intake and IHD.

## Supplementary information

Supplementary information

## Data Availability

The data that support the findings of this study are available from Diet, Cancer and Health Steering Committee at the Danish Cancer Society but restrictions apply to the availability of these data, which were used under license for the current study, and so are not publicly available.
