# Supplementary material for: Intake of dietary flavonoids and incidence of ischemic heart disease in the Danish Diet, Cancer, and Health cohort
Source: Eur J Clin Nutr. 2022 Oct 25;77(2):270–7. doi: 10.1038/s41430-022-01226-y (PMC9908533; doi:10.1038/s41430-022-01226-y)
Supplement: Supplementary file 1 — Supplementary information [file 41430_2022_1226_MOESM1_ESM.docx]

**Supplementary information**

**Intake of dietary flavonoids and incidence of ischemic heart disease in** [**the Danish Diet, Cancer, and Health Cohort**](javascript:void(0))

Parmenter *et al.*

**Participants enrolled between 1993 and 1997 in the Danish Diet, Cancer, and Health study**

(n=57,053)

**Participants excluded** (n=2,557)

- Incomplete Food Frequency Questionnaire (n=51)
- Abnormal energy intake (<500 kcal or >5000 kcal per day) (n=150)
- Missing covariates or extreme outliers (n=211)
- Ischemic heart disease at baseline (n=1557)
- Percutaneous coronary intervention (n = 6)
- Coronary artery bypass graft (n = 8)
- Cancer diagnosis before baseline (n=574)

**21-year follow-up**

(n=54,496)

**Supplementary Figure 1**. Participant flow diagram in the Danish Diet, Cancer, and Health study.

**Supplementary Table 1.** International Classification of Disease codes used to determine prevalent disease

| **Disease and cause specific mortality** | **ICD codes (ICD-8; ICD-10)** |
| --- | --- |
| Ischemic heart disease | 410-414; I20-I25 |
| Hemorrhagic stroke | 43100, 43108, 43109, 43190, 43191, 43198, 43199, 44319; I61 |
| Ischemic stroke | 433-434; I63 |
| Peripheral artery disease | 440-444; I70-I74 |
| Atrial fibrillation | 42793-42794; I48 |
| Chronic kidney disease | 580-584; N02-N08, N11-N12, N14, N18-N19, N26, N158-N160, N162-N164, N168, Q61, E102, E112, E132, E142, I120, M321B |
| Chronic obstructive pulmonary disease | 491-493; J42-J44 |


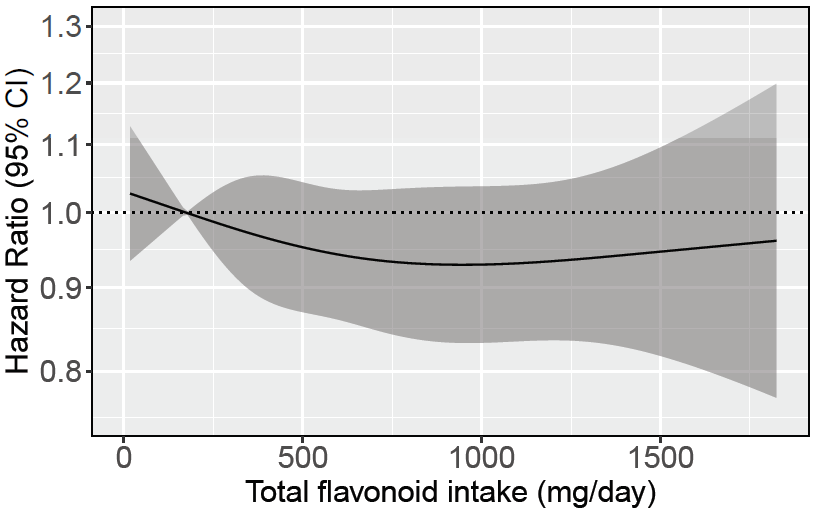


**Supplementary Figure 2.** Cubic spline curve describing the association between total flavonoid intake (mg/day) and validated cases of ischemic heart disease. Hazard ratios are based on Cox proportional hazards models adjusted for age, sex, BMI, smoking status, physical activity, alcohol intake, education, socioeconomic status (income), aspirin use, antihypertensive medication use and statin use and are comparing the specific level of flavonoid intake (horizontal axis) to the median intake for participants in the lowest intake quintile.

| **Supplementary Table 2.** Hazard ratios (95% CIs) of first-time ischemic heart disease by quintiles of flavonoid intake in males and females*^1^* | | | | | | | | | | | |
| --- | --- | --- | --- | --- | --- | --- | --- | --- | --- | --- | --- |
|  |  | Males | | | |  |  | Females | | | |
|  | **Q1** | **Q2** | **Q3** | **Q4** | **Q5** |  | **Q1** | **Q2** | **Q3** | **Q4** | **Q5** |
| **Total Flavonoids** | |  |  |  |  |  |  |  |  |  |  |
| Intake (mg/d)* | 155 (6-223) | 289 (223-354) | 433 (354-539) | 654 (539-807) | 1076 (807-2950) |  | 197 (14-283) | 357 (283-446) | 553 (446-660) | 796 (660-990) | 1291 (990-3552) |
| No. events | 809 | 782 | 724 | 667 | 623 |  | 461 | 402 | 381 | 349 | 362 |
| HR (95% CI) |  |  |  |  |  |  |  |  |  |  |  |
| Model 1 | Ref. | 0.88 (0.83, 0.94) | 0.81 (0.74, 0.87) | 0.75 (0.69, 0.81) | 0.68 (0.63, 0.75) |  | Ref. | 0.84 (0.77, 0.92) | 0.73 (0.66, 0.82) | 0.69 (0.61, 0.77) | 0.75 (0.67, 0.84) |
| Model 2 | Ref. | 0.98 (0.92, 1.04) | 0.96 (0.89, 1.05) | 0.95 (0.87, 1.03) | 0.90 (0.82, 0.99) |  | Ref. | 0.98 (0.90, 1.07) | 0.96 (0.86, 1.07) | 0.96 (0.85, 1.08) | 1.08 (0.95, 1.22) |
| Model 3 | Ref. | 0.99 (0.93, 1.06) | 0.99 (0.90, 1.08) | 0.98 (0.89, 1.07) | 0.94 (0.85, 1.04) |  | Ref. | 0.97 (0.89, 1.06) | 0.95 (0.85, 1.07) | 0.96 (0.85, 1.09) | 1.09 (0.95, 1.24) |
| **Flavonols** | |  |  |  |  |  |  |  |  |  |  |
| Intake (mg/d)* | 14 (0-19) | 24 (19-29) | 35 (29-42) | 57 (42-73) | 104 (73-238) |  | 16 (1-22) | 28 (22-35) | 44 (35-59) | 73 (59-96) | 128 (96-251) |
| No. events | 859 | 764 | 686 | 703 | 593 |  | 491 | 404 | 364 | 359 | 337 |
| HR (95% CI) |  |  |  |  |  |  |  |  |  |  |  |
| Model 1 | Ref. | 0.86 (0.81, 0.91) | 0.78 (0.72, 0.84) | 0.73 (0.67, 0.79) | 0.67 (0.61, 0.73) |  | Ref. | 0.83 (0.77, 0.90) | 0.72 (0.64, 0.80) | 0.65 (0.58, 0.73) | 0.71 (0.63, 0.80) |
| Model 2 | Ref. | 0.95 (0.89, 1.01) | 0.93 (0.85, 1.00) | 0.93 (0.85, 1.02) | 0.89 (0.81, 0.98) |  | Ref. | 0.97 (0.89, 1.05) | 0.94 (0.84, 1.06) | 0.93 (0.82, 1.04) | 1.04 (0.92, 1.18) |
| Model 3 | Ref. | 0.96 (0.90, 1.02) | 0.95 (0.87, 1.03) | 0.97 (0.88, 1.06) | 0.93 (0.84, 1.03) |  | Ref. | 0.96 (0.88, 1.04) | 0.92 (0.82, 1.05) | 0.93 (0.82, 1.05) | 1.06 (0.93, 1.21) |
| **Flavanol monomers** | |  |  |  |  |  |  |  |  |  |  |
| Intake (mg/d)* | 12 (0-18) | 24 (18-34) | 52 (34-83) | 132 (83-268) | 458 (268-886) |  | 16 (0-25) | 38 (25-59) | 102 (59-150) | 268 (150-315) | 488 (315-916) |
| No. events | 865 | 729 | 694 | 709 | 608 |  | 481 | 401 | 367 | 362 | 344 |
| HR (95% CI) |  |  |  |  |  |  |  |  |  |  |  |
| Model 1 | Ref. | 0.91 (0.88, 0.94) | 0.79 (0.72, 0.86) | 0.74 (0.68, 0.8) | 0.70 (0.64, 0.76) |  | Ref. | 0.92 (0.88, 0.97) | 0.77 (0.68, 0.88) | 0.68 (0.6, 0.76) | 0.73 (0.65, 0.82) |
| Model 2 | Ref. | 0.97 (0.93, 1.01) | 0.93 (0.85, 1.02) | 0.93 (0.85, 1.02) | 0.90 (0.82, 0.99) |  | Ref. | 0.99 (0.95, 1.04) | 0.97 (0.86, 1.11) | 0.95 (0.84, 1.07) | 1.01 (0.89, 1.14) |
| Model 3 | Ref. | 0.98 (0.94, 1.02) | 0.96 (0.88, 1.05) | 0.97 (0.89, 1.07) | 0.94 (0.86, 1.04) |  | Ref. | 0.99 (0.95, 1.04) | 0.98 (0.86, 1.12) | 0.96 (0.85, 1.09) | 1.04 (0.92, 1.18) |
| **Flavanol oligo+polymers** | |  |  |  |  |  |  |  |  |  |  |
| Intake (mg/d)* | 82 (0-119) | 159 (119-196) | 233 (196-276) | 332 (276-404) | 506 (404-1991) |  | 105 (5-156) | 199 (156-236) | 277 (236-327) | 386 (327-458) | 558 (458-2254) |
| No. events | 827 | 769 | 738 | 635 | 636 |  | 460 | 435 | 323 | 369 | 368 |
| HR (95% CI) |  |  |  |  |  |  |  |  |  |  |  |
| Model 1 | Ref. | 0.89 (0.83, 0.95) | 0.8 (0.74, 0.87) | 0.72 (0.67, 0.78) | 0.67 (0.62, 0.74) |  | Ref. | 0.79 (0.72, 0.86) | 0.71 (0.64, 0.78) | 0.69 (0.62, 0.76) | 0.73 (0.65, 0.82) |
| Model 2 | Ref. | 1.00 (0.94, 1.07) | 0.96 (0.89, 1.04) | 0.90 (0.83, 0.98) | 0.87 (0.80, 0.96) |  | Ref. | 0.93 (0.85, 1.02) | 0.92 (0.83, 1.02) | 0.95 (0.85, 1.06) | 1.03 (0.91, 1.17) |
| Model 3 | Ref. | 1.02 (0.95, 1.09) | 0.99 (0.91, 1.07) | 0.93 (0.85, 1.02) | 0.90 (0.82, 1.00) |  | Ref. | 0.93 (0.85, 1.02) | 0.92 (0.82, 1.02) | 0.95 (0.84, 1.07) | 1.03 (0.90, 1.18) |
| **Anthocyanins** | |  |  |  |  |  |  |  |  |  |  |
| Intake (mg/d)* | 5 (0-10) | 13 (10-17) | 20 (17-25) | 36 (25-53) | 71 (53-397) |  | 5 (0-9) | 12 (9-17) | 20 (17-24) | 35 (24-53) | 69 (53-305) |
| No. events | 836 | 730 | 648 | 691 | 700 |  | 469 | 354 | 331 | 406 | 395 |
| HR (95% CI) |  |  |  |  |  |  |  |  |  |  |  |
| Model 1 | Ref. | 0.80 (0.75, 0.85) | 0.73 (0.67, 0.79) | 0.76 (0.7, 0.82) | 0.80 (0.73, 0.88) |  | Ref. | 0.76 (0.69, 0.82) | 0.69 (0.61, 0.77) | 0.77 (0.69, 0.86) | 0.91 (0.80, 1.02) |
| Model 2 | Ref. | 0.94 (0.88, 1.01) | 0.93 (0.85, 1.02) | 0.98 (0.9, 1.07) | 1.01 (0.91, 1.11) |  | Ref. | 0.97 (0.88, 1.07) | 0.99 (0.87, 1.13) | 1.09 (0.97, 1.24) | 1.19 (1.04, 1.36) |
| Model 3 | Ref. | 0.94 (0.88, 1.01) | 0.94 (0.85, 1.03) | 1.00 (0.91, 1.09) | 1.03 (0.93, 1.14) |  | Ref. | 0.96 (0.87, 1.06) | 0.97 (0.85, 1.11) | 1.08 (0.95, 1.22) | 1.16 (1.01, 1.34) |
| **Flavanones** | |  |  |  |  |  |  |  |  |  |  |
| Intake (mg/d)* | 4 (0-6) | 9 (6-13) | 17 (13-26) | 32 (26-49) | 71 (49-564) |  | 3 (0-6) | 9 (6-13) | 18 (13-26) | 32 (26-49) | 69 (49-518) |
| No. events | 796 | 730 | 698 | 658 | 723 |  | 447 | 398 | 391 | 339 | 380 |
| HR (95% CI) |  |  |  |  |  |  |  |  |  |  |  |
| Model 1 | Ref. | 0.94 (0.88, 0.99) | 0.88 (0.81, 0.97) | 0.86 (0.79, 0.93) | 0.88 (0.81, 0.97) |  | Ref. | 0.92 (0.85, 0.99) | 0.83 (0.74, 0.94) | 0.75 (0.67, 0.84) | 0.76 (0.67, 0.86) |
| Model 2 | Ref. | 0.99 (0.94, 1.05) | 0.98 (0.89, 1.07) | 0.95 (0.88, 1.04) | 0.97 (0.88, 1.06) |  | Ref. | 1.01 (0.93, 1.09) | 0.98 (0.87, 1.11) | 0.92 (0.82, 1.02) | 0.91 (0.80, 1.03) |
| Model 3 | Ref. | 1.00 (0.95, 1.06) | 0.99 (0.91, 1.09) | 0.97 (0.89, 1.06) | 0.99 (0.90, 1.08) |  | Ref. | 1.00 (0.93, 1.09) | 0.98 (0.86, 1.11) | 0.90 (0.8, 1.01) | 0.88 (0.77, 1.00) |
| **Flavones** | |  |  |  |  |  |  |  |  |  |  |
| Intake (mg/d)* | 2 (0-3) | 4 (3-4) | 5 (4-6) | 7 (6-9) | 11 (9-50) |  | 2 (0-3) | 4 (3-5) | 5 (5-6) | 7 (6-9) | 12 (9-51) |
| No. events | 755 | 732 | 731 | 682 | 705 |  | 436 | 418 | 346 | 340 | 415 |
| HR (95% CI) |  |  |  |  |  |  |  |  |  |  |  |
| Model 1 | Ref. | 0.94 (0.88, 1.00) | 0.89 (0.83, 0.97) | 0.87 (0.80, 0.94) | 0.87 (0.79, 0.95) |  | Ref. | 0.84 (0.77, 0.92) | 0.75 (0.68, 0.84) | 0.71 (0.64, 0.79) | 0.77 (0.68, 0.87) |
| Model 2 | Ref. | 1.02 (0.95, 1.09) | 1.01 (0.93, 1.09) | 0.98 (0.91, 1.07) | 0.96 (0.88, 1.06) |  | Ref. | 0.94 (0.86, 1.03) | 0.89 (0.80, 1.00) | 0.86 (0.77, 0.96) | 0.90 (0.80, 1.02) |
| Model 3 | Ref. | 1.03 (0.96, 1.10) | 1.03 (0.94, 1.12) | 1.01 (0.93, 1.11) | 1.01 (0.91, 1.12) |  | Ref. | 0.95 (0.86, 1.04) | 0.90 (0.80, 1.01) | 0.86 (0.76, 0.97) | 0.88 (0.76, 1.01) |
| ^1^Hazard ratios (95% CI) for first-time ischemic heart disease during 20.8 years of follow-up, obtained from restricted cubic splines in Cox proportional hazards models. Model 1 adjusted for age and sex; Model 2 adjusted for age, sex, BMI, smoking status, physical activity, alcohol intake, education, socioeconomic status (income), aspirin use, antihypertensive medication use and statin use; Model 3 adjusted for all covariates in Model 2 plus intakes of fish, red meat, processed meat, whole grains, processed grains, polyunsaturated fatty acids, monounsaturated fatty acids, saturated fatty acids and energy.  *Median; range in parentheses (all such values). | | | | | | | | | | | |

| **Supplementary Table 3.** Hazard ratios (95% CIs) of first-time ischemic heart disease by quintiles of flavonoid intake in never-smokers and ever-smokers*^1^* | | | | | | | | | | | |
| --- | --- | --- | --- | --- | --- | --- | --- | --- | --- | --- | --- |
|  |  | Never-smokers | | | |  |  | Ever-smokers | | | |
|  | **Q1** | **Q2** | **Q3** | **Q4** | **Q5** |  | **Q1** | **Q2** | **Q3** | **Q4** | **Q5** |
| **Total Flavonoids** | |  |  |  |  |  |  |  |  |  |  |
| Intake (mg/d)* | 215 (8-298) | 373 (298-462) | 570 (462-671) | 798 (671-986) | 1272 (987-2950) |  | 156 (6-228) | 297 (229-366) | 455 (366-563) | 685 (563-859) | 1151 (859-3552) |
| No. events | 289 | 267 | 280 | 259 | 236 |  | 994 | 948 | 823 | 785 | 679 |
| HR (95% CI) |  |  |  |  |  |  |  |  |  |  |  |
| Model 1 | Ref. | 1.00 (0.90, 1.11) | 0.99 (0.86, 1.13) | 0.97 (0.85, 1.12) | 0.96 (0.83, 1.12) |  | Ref. | 0.88 (0.83, 0.94) | 0.79 (0.73, 0.85) | 0.70 (0.65, 0.76) | 0.67 (0.62, 0.73) |
| Model 2 | Ref. | 1.07 (0.96, 1.19) | 1.11 (0.97, 1.28) | 1.13 (0.98, 1.30) | 1.15 (0.99, 1.34) |  | Ref. | 0.98 (0.92, 1.04) | 0.94 (0.87, 1.02) | 0.90 (0.83, 0.97) | 0.89 (0.81, 0.97) |
| Model 3 | Ref. | 1.07 (0.96, 1.20) | 1.12 (0.97, 1.29) | 1.14 (0.98, 1.33) | 1.17 (0.99, 1.38) |  | Ref. | 0.98 (0.93, 1.05) | 0.95 (0.88, 1.03) | 0.91 (0.84, 0.99) | 0.91 (0.83, 1.00) |
| **Flavonols** | |  |  |  |  |  |  |  |  |  |  |
| Intake (mg/d)* | 18 (1-24) | 30 (24-36) | 46 (36-61) | 73 (61-95) | 124 (95-228) |  | 14 (0-19) | 24 (19-29) | 36 (29-45) | 61 (45-78) | 112 (78-251) |
| No. events | 278 | 301 | 259 | 266 | 227 |  | 1062 | 900 | 819 | 777 | 671 |
| HR (95% CI) |  |  |  |  |  |  |  |  |  |  |  |
| Model 1 | Ref. | 1.02 (0.92, 1.13) | 1.01 (0.88, 1.17) | 0.96 (0.83, 1.10) | 0.91 (0.79, 1.06) |  | Ref. | 0.84 (0.80, 0.89) | 0.74 (0.69, 0.80) | 0.67 (0.62, 0.72) | 0.65 (0.60, 0.71) |
| Model 2 | Ref. | 1.10 (0.99, 1.21) | 1.16 (1.00, 1.34) | 1.13 (0.98, 1.30) | 1.11 (0.95, 1.29) |  | Ref. | 0.93 (0.88, 0.98) | 0.89 (0.82, 0.96) | 0.87 (0.80, 0.94) | 0.87 (0.79, 0.95) |
| Model 3 | Ref. | 1.09 (0.99, 1.22) | 1.15 (0.99, 1.35) | 1.14 (0.98, 1.33) | 1.12 (0.95, 1.32) |  | Ref. | 0.93 (0.88, 0.99) | 0.90 (0.83, 0.97) | 0.89 (0.81, 0.97) | 0.90 (0.82, 0.99) |
| **Flavanol monomers** | |  |  |  |  |  |  |  |  |  |  |
| Intake (mg/d)* | 18 (0-27) | 42 (27-63) | 111 (63-255) | 268 (255-305) | 485 (305-886) |  | 12 (0-18) | 26 (18-37) | 56 (37-98) | 250 (98-274) | 467 (274-916) |
| No. events | 288 | 276 | 292 | 241 | 234 |  | 1082 | 890 | 794 | 793 | 670 |
| HR (95% CI) |  |  |  |  |  |  |  |  |  |  |  |
| Model 1 | Ref. | 1.03 (0.97, 1.10) | 1.08 (0.92, 1.26) | 0.97 (0.85, 1.12) | 0.93 (0.80, 1.07) |  | Ref. | 0.90 (0.87, 0.93) | 0.77 (0.71, 0.83) | 0.68 (0.63, 0.74) | 0.69 (0.64, 0.75) |
| Model 2 | Ref. | 1.06 (1.00, 1.13) | 1.17 (1.00, 1.38) | 1.12 (0.97, 1.30) | 1.08 (0.93, 1.25) |  | Ref. | 0.96 (0.93, 1.00) | 0.91 (0.84, 0.99) | 0.89 (0.81, 0.96) | 0.89 (0.82, 0.97) |
| Model 3 | Ref. | 1.07 (1.00, 1.14) | 1.19 (1.01, 1.40) | 1.15 (0.99, 1.33) | 1.10 (0.95, 1.28) |  | Ref. | 0.97 (0.93, 1.00) | 0.93 (0.85, 1.01) | 0.91 (0.84, 1.00) | 0.92 (0.85, 1.01) |
| **Flavanol oligo+polymers** | |  |  |  |  |  |  |  |  |  |  |
| Intake (mg/d)* | 115 (1-163) | 204 (163-242) | 281 (242-331) | 387 (331-459) | 561 (459-1991) |  | 83 (0-123) | 166 (123-204) | 241 (204-288) | 344 (288-418) | 521 (418-2254) |
| No. events | 294 | 293 | 233 | 250 | 261 |  | 1025 | 942 | 807 | 755 | 700 |
| HR (95% CI) |  |  |  |  |  |  |  |  |  |  |  |
| Model 1 | Ref. | 0.95 (0.85, 1.05) | 0.92 (0.81, 1.04) | 0.91 (0.80, 1.04) | 0.94 (0.81, 1.08) |  | Ref. | 0.87 (0.82, 0.93) | 0.77 (0.72, 0.83) | 0.69 (0.64, 0.74) | 0.67 (0.61, 0.72) |
| Model 2 | Ref. | 1.03 (0.92, 1.14) | 1.04 (0.91, 1.18) | 1.05 (0.92, 1.21) | 1.11 (0.96, 1.29) |  | Ref. | 0.98 (0.92, 1.04) | 0.93 (0.87, 1.00) | 0.87 (0.80, 0.94) | 0.87 (0.80, 0.94) |
| Model 3 | Ref. | 1.04 (0.93, 1.16) | 1.05 (0.92, 1.20) | 1.06 (0.92, 1.23) | 1.13 (0.96, 1.33) |  | Ref. | 0.99 (0.93, 1.05) | 0.94 (0.88, 1.02) | 0.88 (0.81, 0.96) | 0.88 (0.80, 0.97) |
| **Anthocyanins** | |  |  |  |  |  |  |  |  |  |  |
| Intake (mg/d)* | 6 (0-10) | 13 (10-17) | 20 (17-24) | 31 (24-52) | 63 (52-305) |  | 5 (0-9) | 12 (9-17) | 20 (17-25) | 38 (25-54) | 80 (54-397) |
| No. events | 283 | 252 | 238 | 278 | 280 |  | 989 | 853 | 761 | 814 | 812 |
| HR (95% CI) |  |  |  |  |  |  |  |  |  |  |  |
| Model 1 | Ref. | 0.87 (0.78, 0.97) | 0.83 (0.72, 0.95) | 0.86 (0.75, 0.98) | 0.95 (0.82, 1.11) |  | Ref. | 0.79 (0.75, 0.84) | 0.71 (0.66, 0.77) | 0.75 (0.69, 0.81) | 0.80 (0.74, 0.87) |
| Model 2 | Ref. | 0.98 (0.87, 1.10) | 0.98 (0.84, 1.15) | 1.04 (0.89, 1.2) | 1.12 (0.95, 1.33) |  | Ref. | 0.96 (0.9, 1.02) | 0.95 (0.87, 1.04) | 1.03 (0.94, 1.12) | 1.05 (0.95, 1.14) |
| Model 3 | Ref. | 0.97 (0.86, 1.09) | 0.97 (0.83, 1.14) | 1.03 (0.89, 1.2) | 1.12 (0.94, 1.33) |  | Ref. | 0.96 (0.9, 1.02) | 0.96 (0.88, 1.05) | 1.03 (0.95, 1.13) | 1.05 (0.96, 1.16) |
| **Flavanones** | |  |  |  |  |  |  |  |  |  |  |
| Intake (mg/d)* | 4 (0-7) | 11 (7-14) | 20 (14-27) | 35 (27-51) | 70 (51-483) |  | 3 (0-6) | 9 (6-12) | 16 (12-25) | 31 (25-48) | 70 (48-564) |
| No. events | 279 | 278 | 258 | 234 | 282 |  | 928 | 843 | 850 | 792 | 816 |
| HR (95% CI) |  |  |  |  |  |  |  |  |  |  |  |
| Model 1 | Ref. | 0.98 (0.88, 1.08) | 0.94 (0.81, 1.10) | 0.89 (0.77, 1.02) | 0.92 (0.79, 1.06) |  | Ref. | 0.94 (0.89, 0.99) | 0.88 (0.81, 0.96) | 0.83 (0.77, 0.90) | 0.85 (0.78, 0.92) |
| Model 2 | Ref. | 0.99 (0.90, 1.10) | 0.97 (0.83, 1.13) | 0.92 (0.80, 1.05) | 0.94 (0.81, 1.09) |  | Ref. | 1.00 (0.95, 1.05) | 0.99 (0.91, 1.08) | 0.97 (0.89, 1.04) | 0.98 (0.90, 1.07) |
| Model 3 | Ref. | 0.99 (0.90, 1.10) | 0.97 (0.83, 1.13) | 0.92 (0.80, 1.06) | 0.94 (0.80, 1.10) |  | Ref. | 1.01 (0.96, 1.06) | 1.00 (0.92, 1.09) | 0.97 (0.89, 1.05) | 0.97 (0.89, 1.06) |
| **Flavones** | |  |  |  |  |  |  |  |  |  |  |
| Intake (mg/d)* | 3 (0-3) | 4 (3-5) | 6 (5-7) | 8 (7-9) | 12 (9-44) |  | 2 (0-3) | 4 (3-4) | 5 (4-6) | 7 (6-9) | 11 (9-51) |
| No. events | 290 | 262 | 243 | 257 | 279 |  | 863 | 903 | 832 | 799 | 832 |
| HR (95% CI) |  |  |  |  |  |  |  |  |  |  |  |
| Model 1 | Ref. | 0.88 (0.79, 0.98) | 0.85 (0.75, 0.96) | 0.87 (0.76, 0.99) | 0.93 (0.80, 1.07) |  | Ref. | 0.95 (0.89, 1.01) | 0.89 (0.83, 0.97) | 0.84 (0.78, 0.91) | 0.86 (0.79, 0.93) |
| Model 2 | Ref. | 0.92 (0.83, 1.02) | 0.9 (0.79, 1.02) | 0.91 (0.80, 1.03) | 0.95 (0.82, 1.10) |  | Ref. | 1.02 (0.96, 1.09) | 1.01 (0.94, 1.09) | 0.97 (0.90, 1.05) | 0.97 (0.89, 1.05) |
| Model 3 | Ref. | 0.93 (0.83, 1.04) | 0.92 (0.8, 1.05) | 0.93 (0.80, 1.07) | 0.97 (0.82, 1.15) |  | Ref. | 1.03 (0.97, 1.10) | 1.02 (0.94, 1.11) | 0.98 (0.90, 1.06) | 0.98 (0.89, 1.08) |
| ^1^Hazard ratios (95% CI) for first-time ischemic heart disease during 20.8 years of follow-up, obtained from restricted cubic splines in Cox proportional hazards models. Model 1 adjusted for age and sex; Model 2 adjusted for age, sex, BMI, smoking status, physical activity, alcohol intake, education, socioeconomic status (income), aspirin use, antihypertensive medication use and statin use; Model 3 adjusted for all covariates in Model 2 plus intakes of fish, red meat, processed meat, whole grains, processed grains, polyunsaturated fatty acids, monounsaturated fatty acids, saturated fatty acids and energy. Ever-smokers were additionally adjusted for pack-years of smoking duration.  *Median; range in parentheses (all such values). | | | | | | | | | | | |
